# Supplementary material for: Differential effects of sex and age on daily and infradian rhythms of mice
Source: J Physiol. 2026 Feb 4;604(5):1915–43. doi: 10.1113/JP289676 (PMC12953017; doi:10.1113/JP289676)
Supplement: Supplementary file 3 — Supplementary Figure 1. Age‐related differences in the distribution of locomotor activity across the 24 h light–dark cycle under small‐cage housing conditions. Supplementary Figure 2. Oestrous cycles over 15 consecutive days in young and middle‐aged female mice. Supplementary Figure 3. Sex‐ and age‐related differences in 5 day wheel‐running rhythms. Supplementary Figure 4. Infradian (5 day periodicity) in daily wheel‐running activity levels. Supplementary Figure 5. Example of Lomb‐Scargle periodogram analysis in two mice (young male and young female). Supplementary Figure 6. Relationship between 5 day rhythm strength and onset/offset variance in young and middle‐aged female mice. Supplementary Figure 7. Sex‐related differences in 10 day wheel‐running rhythms. Supplementary Figure 8. Effect of cage change on daily and infradian wheel‐running rhythms of male mice housed in small cages. Supplementary Figure 9. Food and water consumption during the first 10 days of transfer to single‐housing conditions. [file TJP-604-1915-s002.docx]

**Title: Differential Effects of Sex and Age on Daily and Infradian Rhythms of Mice**

Pishan Chang^1*^, Timna Hitrec^1,2^, Charlotte Muir^1^, Meida Sofyana^1,3^, Vuong Hung Truong^4^, Shannon Lacey^1^, Lukasz Chrobok^1^, Jihwan Myung^4^, Hugh D. Piggins^1*^

1. School of Physiology, Pharmacology & Neuroscience, University of Bristol, University Walk, Bristol, BS8 1TD, UK

2. Department of Biomedical and Neuromotor Sciences, Università di Bologna, Bologna, Italy

3. Department of Physiology, Faculty of Medicine, Public Health and Nursing, Universitas Gadjah Mada, Yogyakarta, Indonesia

4. Graduate Institute of Mind Brain and Consciousness, Taipei Medical University, New Taipei City, Taiwan.

*Corresponding authors:

Dr. Pishan Chang, School of Physiology, Pharmacology and Neuroscience, Biomedical Sciences Building, University of Bristol, University Walk, Bristol, BS8 1TD, UK. Email: [pishan.chang@bristol.ac.uk](mailto:pishan.chang@bristol.ac.uk).

Prof. Hugh D. Piggins, School of Physiology, Pharmacology and Neuroscience, Biomedical Sciences Building, University of Bristol, University Walk, Bristol, BS8 1TD, UK. Email: [hugh.piggins@bristol.ac.uk](mailto:hugh.piggins@bristol.ac.uk).


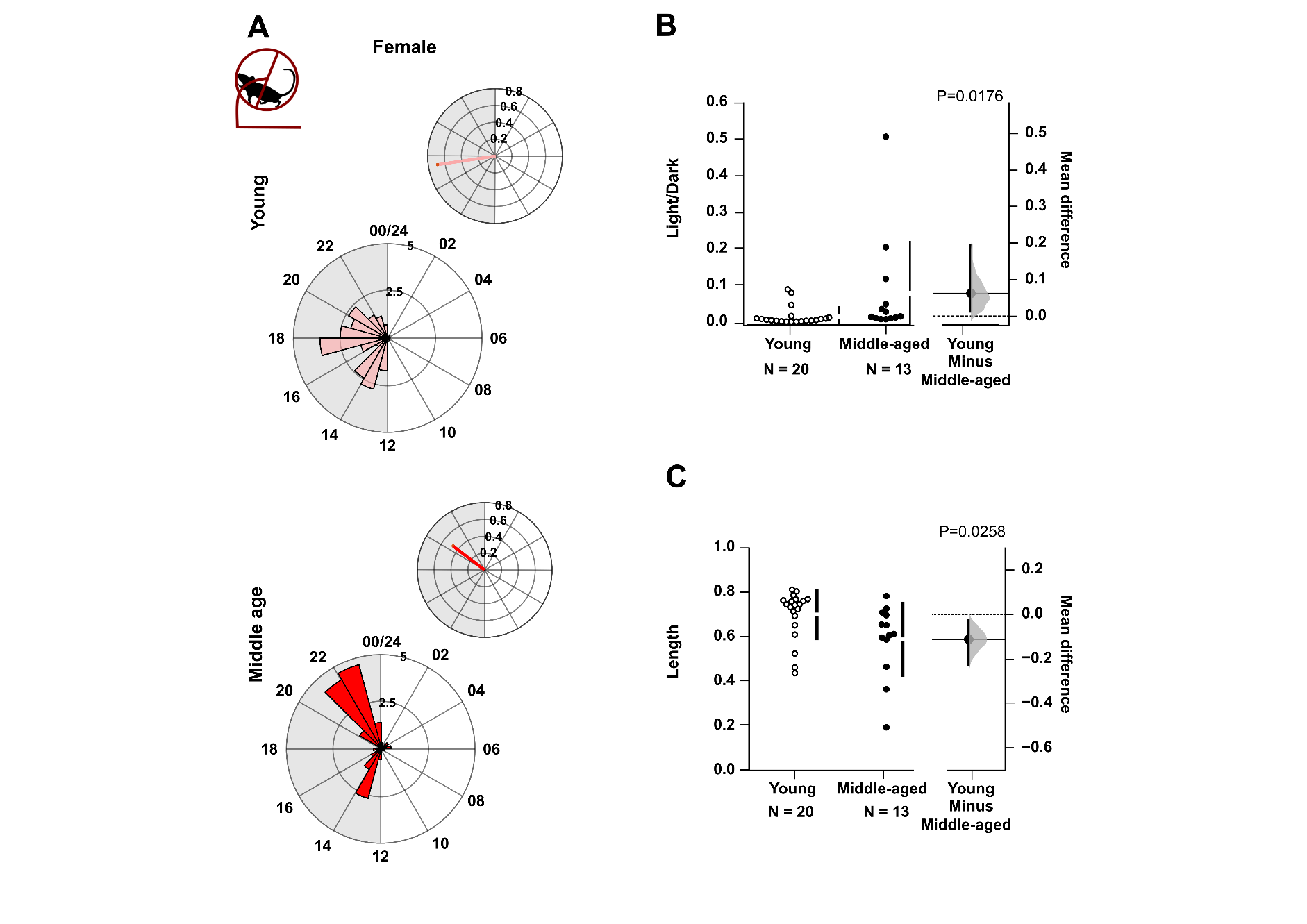


**Supplementary Figure 1. Age-related differences in the distribution of locomotor activity across the 24-hour light-dark cycle under small-cage housing conditions.**

**(A)** Representative daily wheel-running activity in young and middle-aged female mice, shown as rose plots aligned to time of day (0-24 h), averaged over a 10-day recording period. Each petal represents a 1-hour bin; the petal height reflects mean activity. The upper panel includes the mean resultant vector, where direction indicates peak activity time and length indicates its rhythmic regularity. **(B)** Comparison of the light-to-dark phase activity ratio between age groups (male and female animals combined). **(C)** Comparison of vector lengths (regularity of peak) across age groups (male and female animals combined). For **(B)** and **(C)**, raw data are shown on the left; bootstrap sampling distributions of mean differences are on the right. Dots represent mean differences; vertical lines show 95% confidence intervals. Permutation *t*-tests were performed with 5,000 bootstrap samples; confidence intervals are bias-corrected and accelerated. Reported p-values reflect the probability of observing the effect size (or greater) under the null hypothesis. Full statistical details are available in the supplementary file. Animals identified as outliers (Grubbs' test) were excluded from the analysis.

**
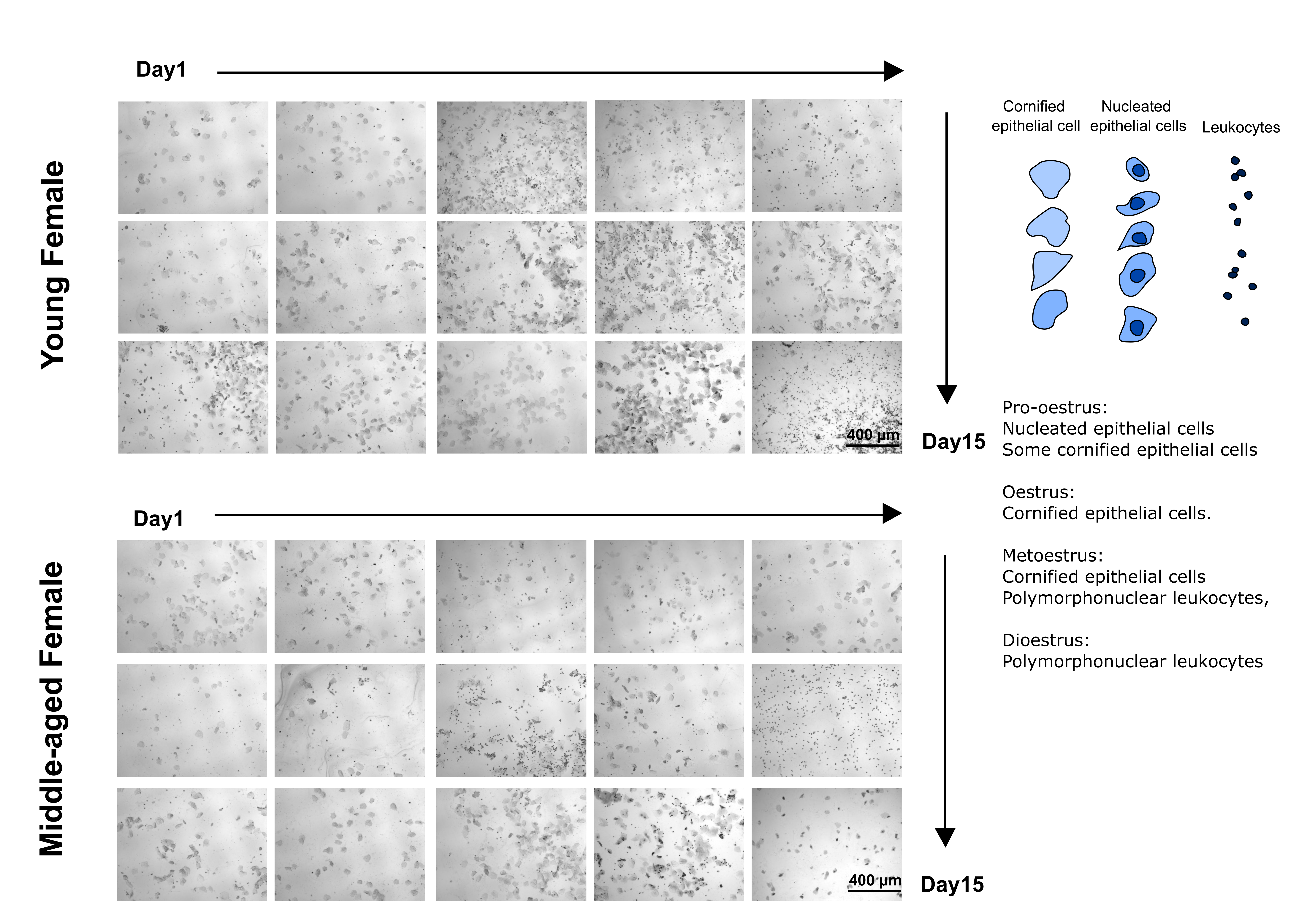
**

**Supplementary Figure 2. Oestrous cycle over 15 consecutive days in young and middle-aged female mice.**

Representative cytological samples collected daily from vaginal smears illustrate the progression of the oestrous cycle in a young but not middle-aged female mouse. The visual representation shows the relative proportion and morphology of predominant cell types, nucleated epithelial cells, cornified epithelial cells, and leukocytes, across the four canonical stages: pro-oestrus, oestrus, metoestrus, and dioestrus. This young female mouse which showed 5-day rhythms in behaviour also exhibited regular oestrous cycling with a clear 4-6 day periodicity in vaginal cytology. In contrast, a middle-aged female mouse that did not express 5-day rhythms in behaviour showed irregular and less distinguishable patterns, indicating age-related disruption in reproductive cycling.


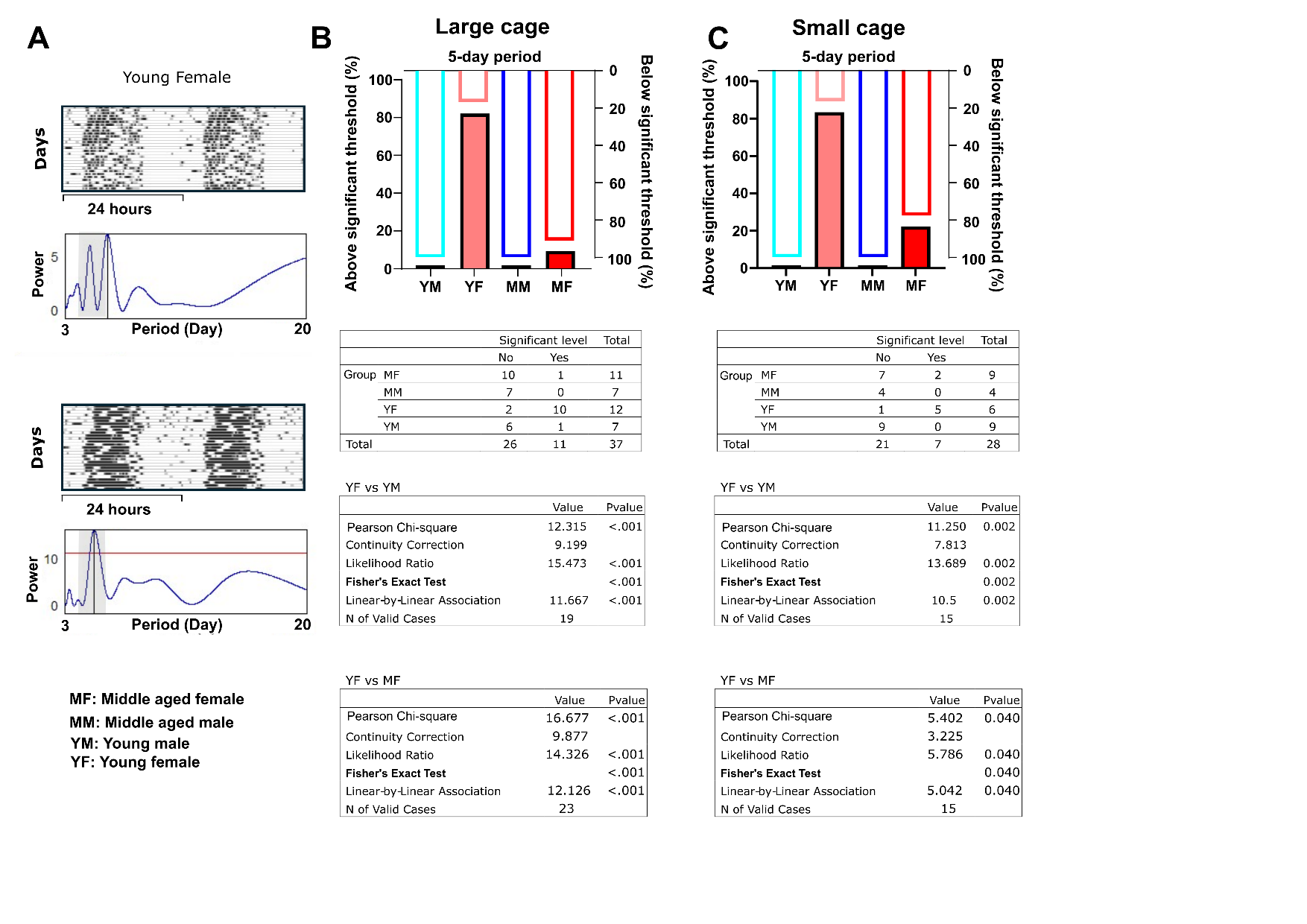


**Supplementary Figure 3. Sex- and age-related differences in 5-day wheel-running rhythms.**

The presence and significance of infradian rhythms of 5-day periodicity in locomotor (wheel-running) activity differs across sex and age groups. **(A)** Example actogram and periodogram (period range: 3-20 days) from a young female mouse. The periodogram shows peaks above and below the significance threshold (*p* < 0.05). The grey shaded area highlights the 5 ± 1 day period, and the red line indicates the significance threshold. **(B)** Summary of the proportion of animals with significant 5-day rhythms across sex and age groups of mice recorded in large cages. **(C)** Summary of the proportion of animals with significant 5-day rhythms across sex and age groups recorded in small cages. Only mice subjected to the 10-day cage change schedule were included for analysis. The top panel displays the percentage of animals whose rhythm strength reaches significance. The middle table summarises statistical comparisons between young females and young males. The bottom table shows comparisons between young and middle-aged female mice.


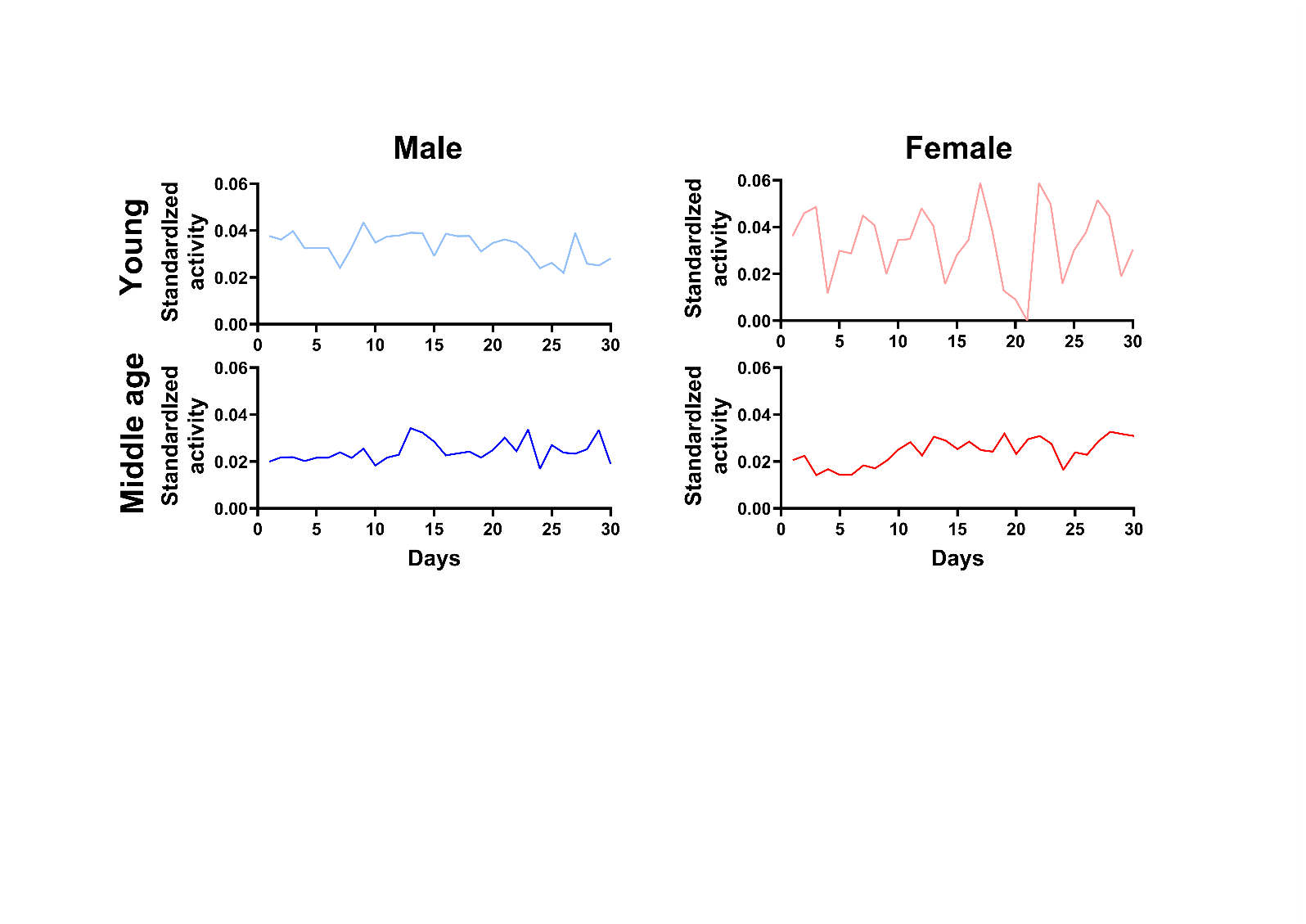
**Supplementary Figure 4. Infradian (5-day periodicity) in daily wheel-running activity levels.**

Top right: representative plot showing the summed daily wheel-running activity of a young female mouse across the recording epoch, demonstrating a clear 5-day infradian rhythm (highlighted by recurring peaks in daily wheel-running). In contrast, this pattern was not observed in representative profiles from young male (top left), middle-aged male (bottom left), or middle-aged female (bottom right) mice. In these mice, wheel-running activity patterns appeared more variable and lacked consistent 5-day periodicity. These examples highlight a sex- and age-specific expression of 5-day rhythms in locomotor behaviour.


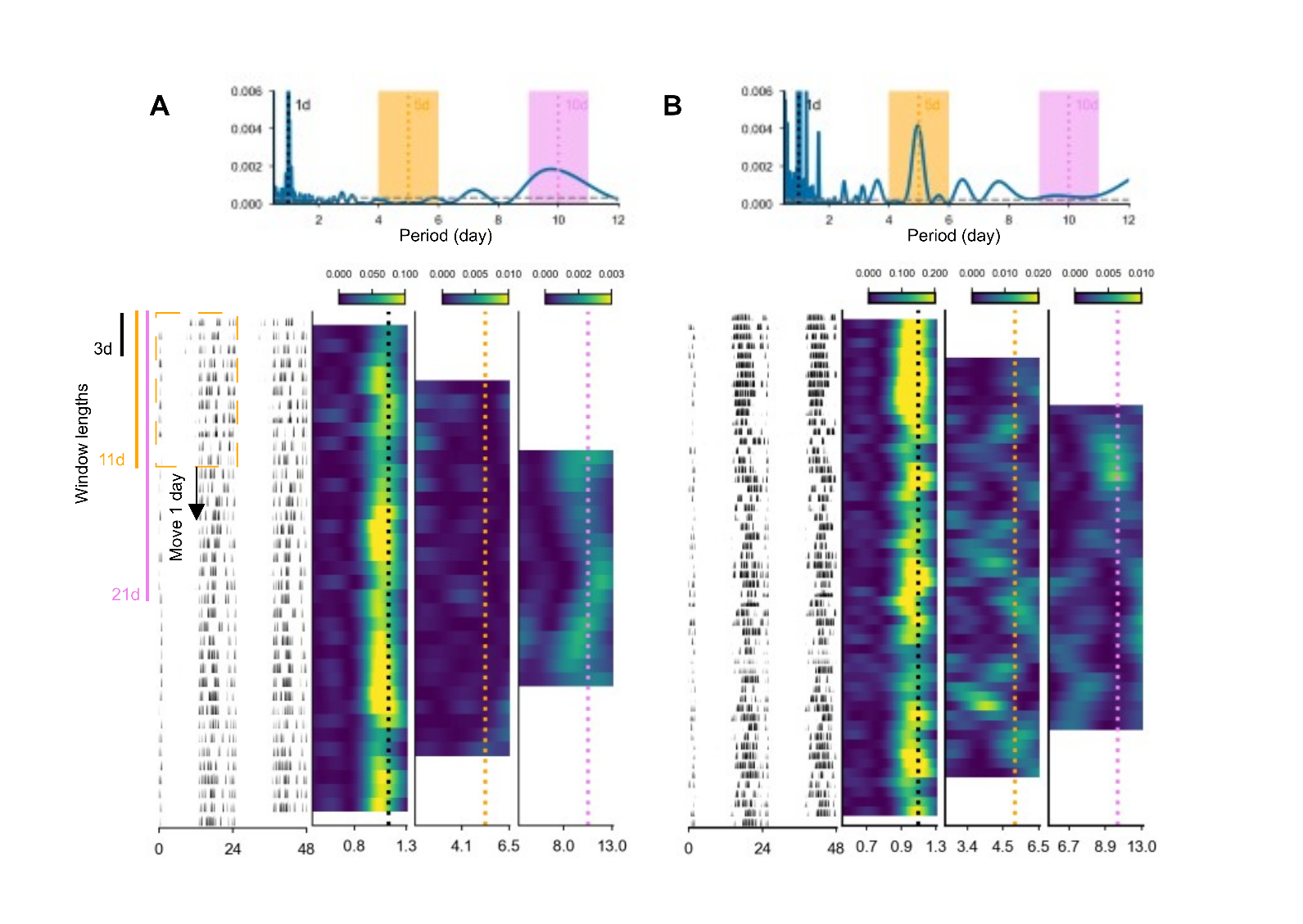


**Supplementary Figure 5. Example of Lomb-Scargle periodogram analysis in two mice (young male and young female).**

Lomb-Scargle (LS) periodogram analyses for young male **(A)** and young female **(B)** mouse are shown. Upper panel: LS power spectrum of the entire dataset, revealing significant period peaks at approximately 1 day, 5 days, and 13 days. The required peak height to achieve a false alarm probability (FAP) of 0.05 is indicated by the dashed gray line. Shaded areas represented the ±1 day range. Lower panel: Left: Double-plot of wheel-running activity across 38 days **(A)**, and 55 days **(B)** plotted on a 48-hour axis to visualize rhythmic patterns. Right: Moving LS periodogram using varying window lengths, showing rhythms at different temporal scales: 0.6-1.3 days (circadian), 3-6.5 days (~5-day), and 6-13 days (~10-day). Periodograms were calculated within sliding windows of 3 (dashed black line), 11 (dashed orange line), or 21 (dashed pink line) days, each advancing in 1-day increments. Notably, although the full-periodogram (Upper panel) indicates clear rhythmic peaks, the moving periodogram reveals that these rhythms may not be stable across the entire recording period (Lower panel: 3-6.5 days and 6-13 days spectrogram). Missing data was imputed by the scaled average of the two previous days.


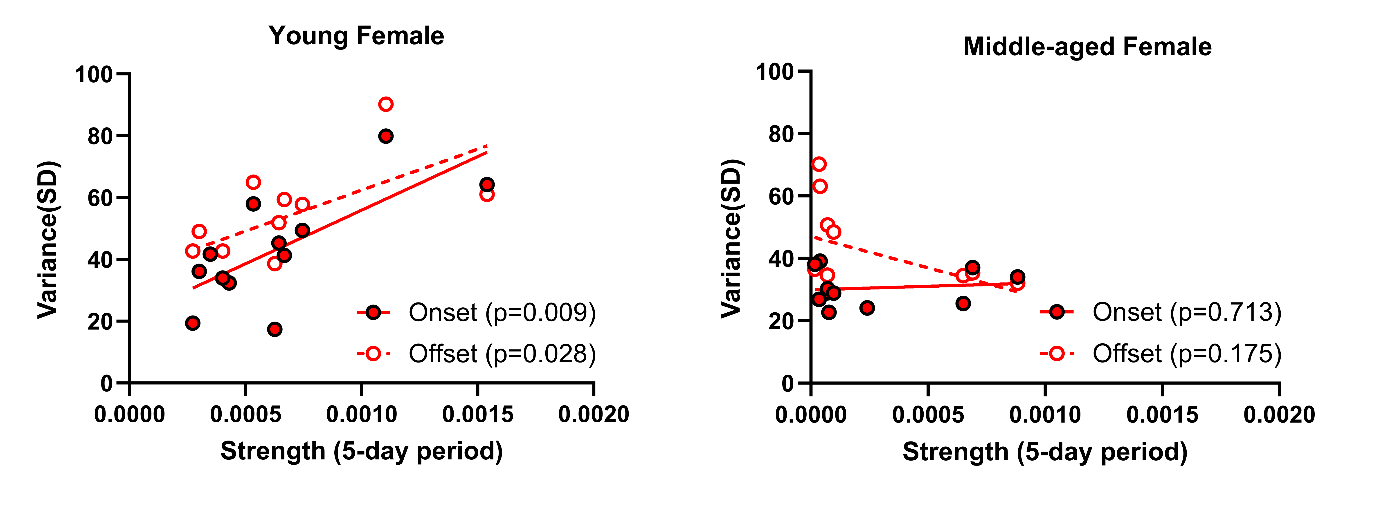
**Supplementary Figure 6. Relationship between 5-day rhythm strength and onset/offset variance in young and middle-aged female mice.**

**(A)** Scatter plot showing the correlation between onset and offset variance in young female mice, reflecting the consistency of daily timing in this age group. **(B)** Scatter plot showing the correlation between onset and offset variance in middle-aged female mice, highlighting age-related changes in the stability of daily rhythms. Each point represents an individual animal; lines represent linear regression fits. Pearson correlation coefficients were used to assess the strength and direction of the associations, with statistically significant correlations indicated by corresponding p-values. Young female (n=12); Middle aged female (n=11). For statistical details, please refer to the supplementary statistical summary file.


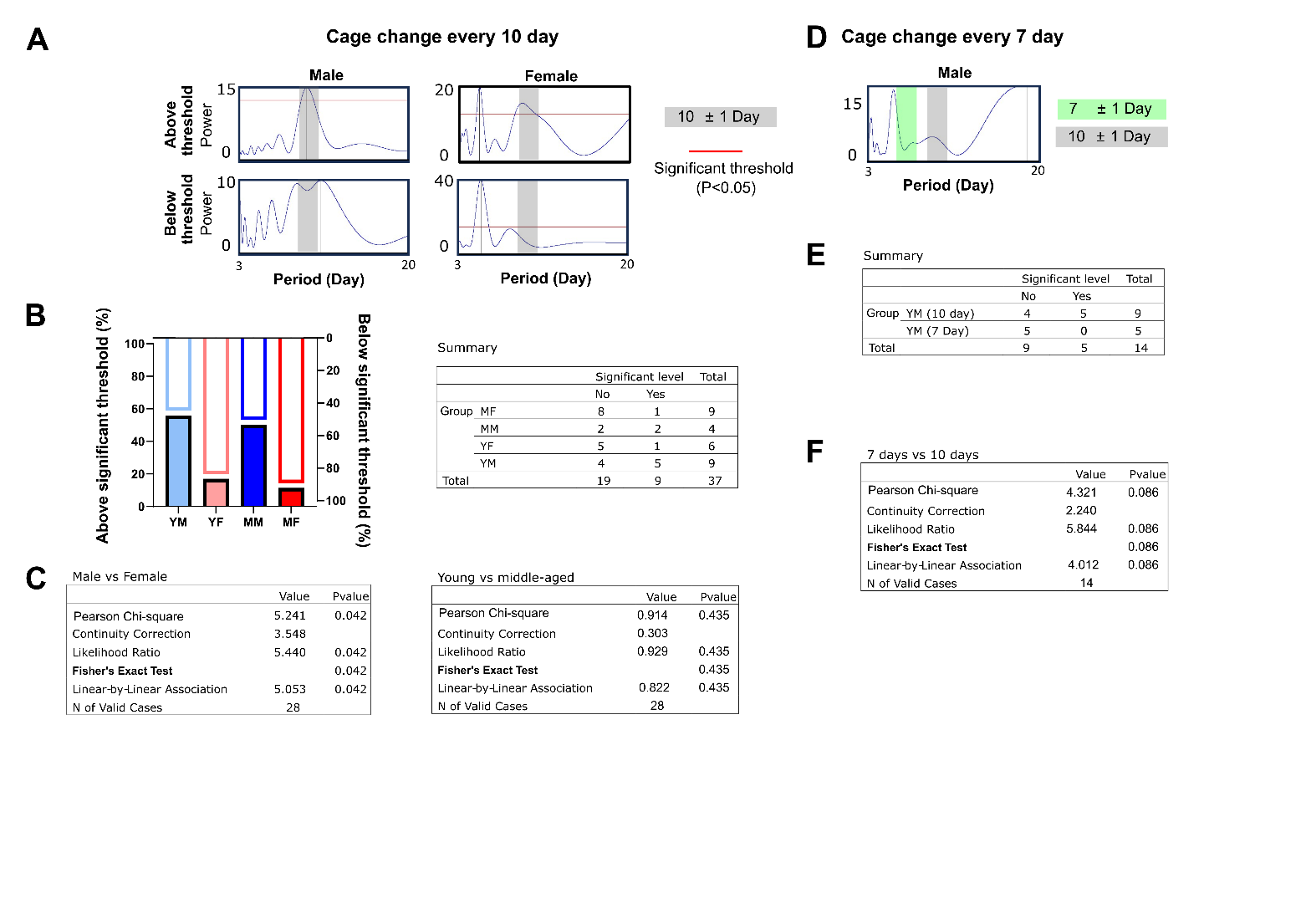
**Supplementary Figure 7. Sex -related differences in 10-day wheel-running rhythms.**

This figure illustrates sex and age-related differences in 10-day infradian rhythms in wheel-running activity. **(A)** Example actograms and corresponding periodograms (period range: 3-20 days) from a young male and a young female mouse. Peaks above the red significance threshold line (*p* < 0.05) indicate significant periodicities. The grey shaded area marks the 10 ± 1 day range. **(B)** Summary of the proportion of animals showing **(C)** significant 10-day rhythms, analyzed by sex in small-cage housing conditions. Comparison of the same data grouped by age shows no age-dependent differences in 10-day rhythm prevalence. **(D)** Example actogram and periodogram from a young male mouse housed with a 7-day cage-change routine. The green shaded area highlights the 7 ± 1 day period, and the grey area again indicates the 10 ± 1 day range. **(E)** Summary of the proportion of animals showing **(F)** no significant difference in the number of animals expressing significant 10-day rhythms across the two cage-change frequency routines.


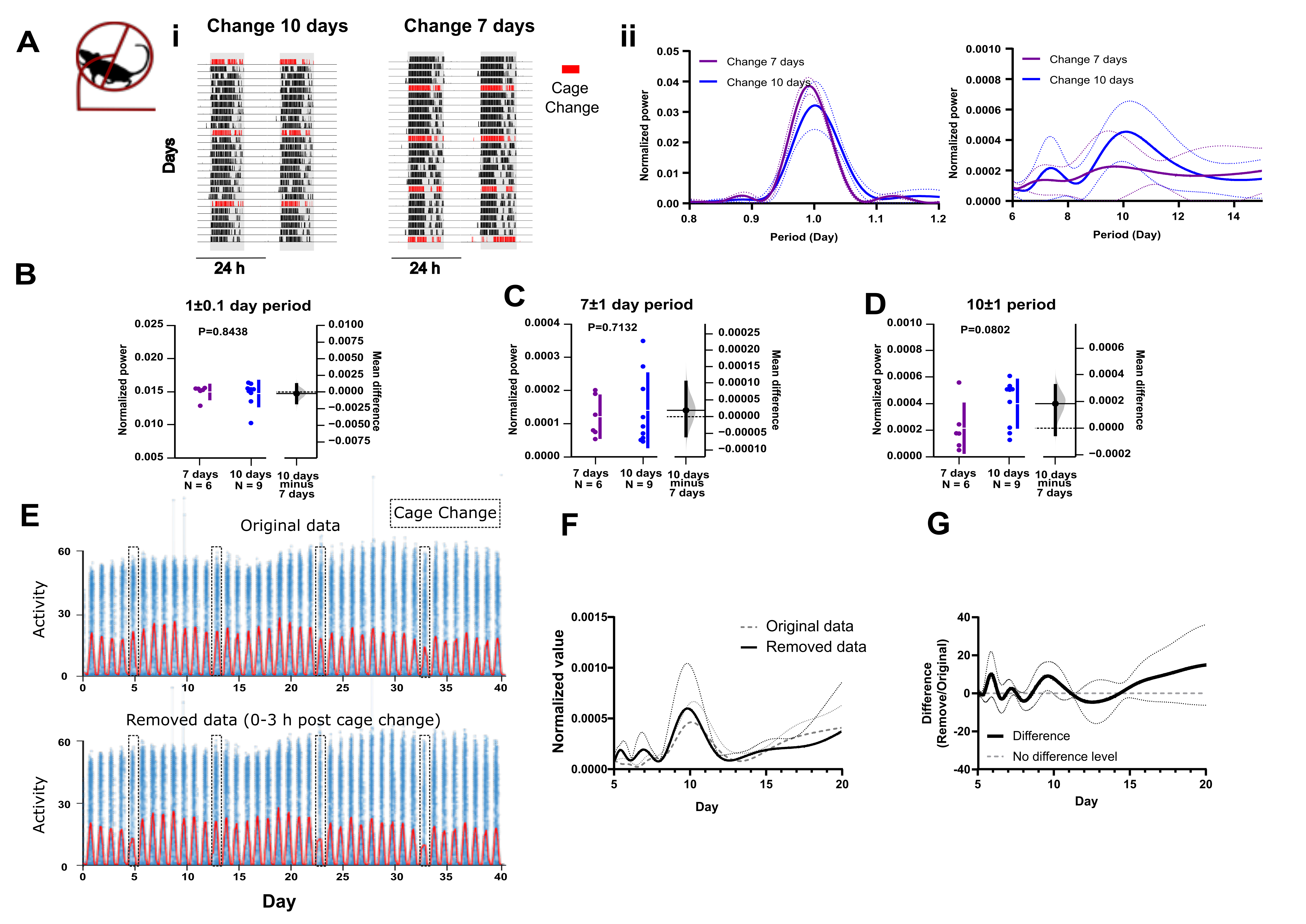


**Supplementary Figure 8. Effect of cage change on daily and infradian wheel-running rhythms of male mice housed in small cages**

**(A)** (i). Example actograms are shown for mice housed under cage-change schedules of every 7 days and every 10 days. (ii) Daily (0.8-1.2 days) and infradian (3-15 days) rhythms in locomotor activity of male mice were analysed and periodograms generated using the Lomb-Scargle method. Data are shown for two conditions: cage change every 7 days and every 10 days. Left: daily rhythm; Right: infradian rhythms. Data are presented as mean (solid line) ± SD (dashed line). **(B)** Comparison of the mean power of 1 ± 0.1 day period between groups. **(C)** Comparison of the mean power of 7 ± 1 day period between groups. **(D)** Comparison of the mean power of 10 ± 1 day period between groups. The mean differences are visualised using Gardner-Altman estimation plots. Both groups are plotted on the left axes, while the mean difference is displayed on a floating axis on the right as a bootstrap sampling distribution. The mean difference is represented by a black-filled dot, with the 95% confidence interval indicated by the vertical error bar. Permutation t-tests were used to assess statistical significance, with p-values reflecting the likelihood of observing the effect size (or greater) under the null hypothesis of zero difference. Sample sizes: *n* = 6 (cage change every 7 days); *n* = 9 (cage change every 10 days). **(E)** Example of daily activity over 40 days (top) and after removing 3 h post-cage change (bottom). Blue dots show original data; red line shows smoothed data. **(F)** LS periodogram (mean ± SD) before (black) and after removal (grey). **(G)** Difference between original and removal data (black, mean ± SD); 0 indicates no change (grey dashed line). Further statistical details are available in the supplementary statistics file.


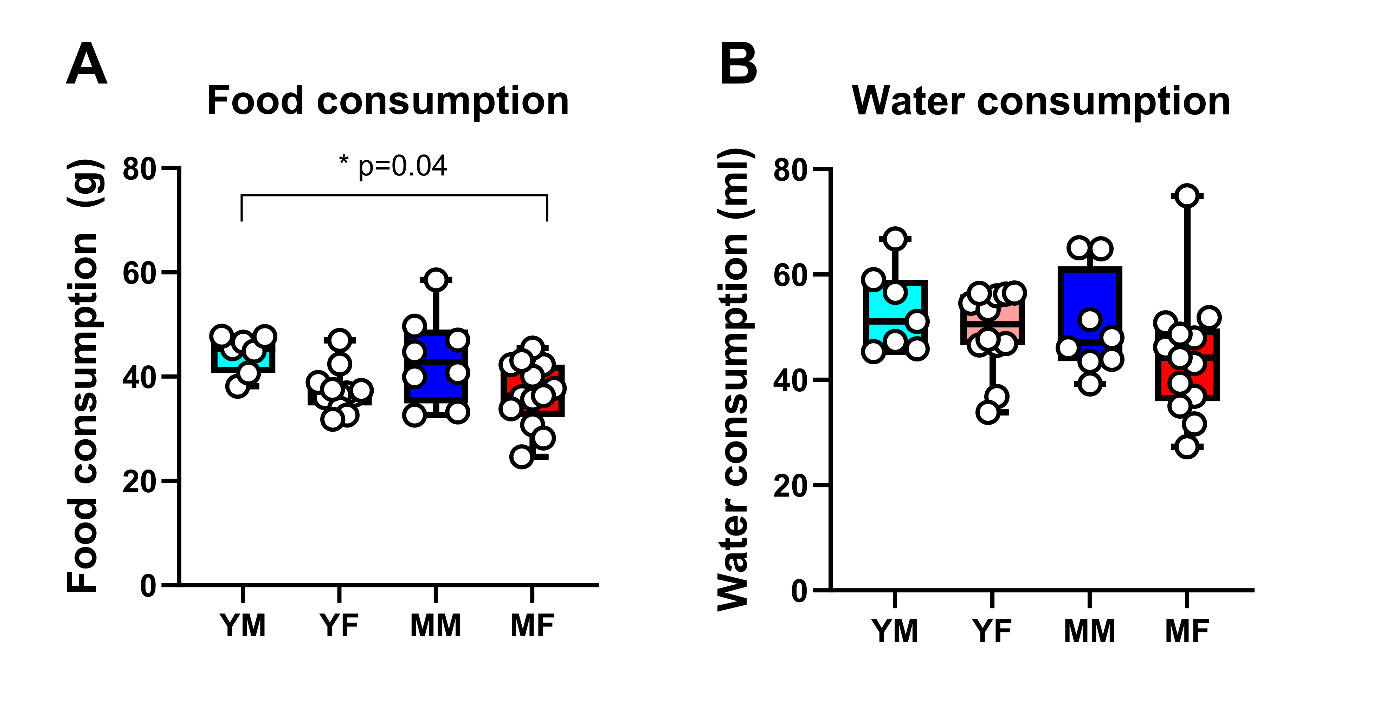
**Supplementary Figure 9. Food and water consumption during the first 10 days of transfer to single-housing conditions.**

(A) Food consumption (grams) and (B) water consumption (millilitres) recorded over the initial 10-day period of single housing in young and middle-aged male and female mice. Data are displayed as box-and-whisker plots showing minimum and maximum values. A significant group effect was observed in food consumption (one-way ANOVA: *F*_(3, 36)_ = 4.344, *p* = 0.0103), with post hoc analysis revealing a significant difference between young male (YM) and middle-aged female (MF) mice (*p* = 0.04). No significant group differences were found in water consumption. Further statistical details are provided in the supplementary file. YM = young male mice (n=7); YF = young female mice (n=10); MM = middle-aged male mice (n=8); MF = middle-aged female mice (n=13). Further statistical details are available in the supplementary statistics file.
